# Mapping the economics of extracorporeal membrane oxygenation: Geographic variation in U.S. reimbursement patterns

**DOI:** 10.1016/j.jhlto.2026.100640

**Published:** 2026-07-18

**Authors:** Priya Joshi, Aryan Gupta, M. Abul Kashem, Roh Yanagida, Parag Desai, Yoshiya Toyoda, Suyog Mokashi

**Affiliations:** a Division of Cardiovascular Surgery, Lewis Katz School of Medicine at Temple University, Philadelphia, Pennsylvania; b Division of Cardiovascular Surgery, Temple University Hospital, Philadelphia, Pennsylvania; c Department of Pulmonary and Thoracic Medicine, Temple University Hospital, Philadelphia, Pennsylvania

**Keywords:** extracorporeal membrane oxygenation (ECMO), health care economics, medicare reimbursement, geographic variation, healthcare disparities, gross domestic product (GDP), resource allocation

## Abstract

We sought to examine the relationship between extra-corporeal membrane oxygenation (ECMO) reimbursement and regional economic performance. Significant variation was observed across states in average payment per discharge, (*p* < 0.0001). The highest mean Medicare payments per discharge occurred in New Hampshire ($21,045), while the lowest was in Oklahoma ($5,487). Gross domestic product (GDP) demonstrated a weak positive correlation with average payment per discharge (*r* = 0.18, *p* = 0.0087), indicating GDP may not be the primary driver of reimbursement. Substantial geographic variation in ECMO reimbursement persists across the U.S., with Medicare payments per discharge varying more than threefold between states.

Extracorporeal membrane oxygenation (ECMO) has become an indispensable therapeutic modality for managing severe, refractory cardiopulmonary failure. As the utilization of ECMO continues to rise, particularly as a bridge to heart transplantation or ventricular assist device (VAD) implantation, the financial implications for healthcare systems have come under increased scrutiny.^1^ The resource-intensive nature of ECMO, including requirements for specialized equipment, multidisciplinary critical care, and prolonged hospital stays, places a substantial economic burden on institutions.^2^

Recent literature has increasingly highlighted profound heterogeneity in healthcare reimbursement patterns across the United States, particularly within cardiothoracic surgery.^3^ Despite standardized national billing frameworks such as the Medicare Severity Diagnosis-Related Group (MS-DRG) system, hospital compensation for high-acuity interventions remains inconsistent.^4^ While regional cost-of-living differences are often assumed to drive these variations,^3^ the impact of local economic performance on ECMO reimbursement remains poorly characterized.

This study sought to examine the relationship between ECMO reimbursement and regional economic performance. Specifically, we aimed to determine whether differences in metropolitan Gross Domestic Product (GDP) account for the geographic disparities observed between billed hospital charges and realized Medicare payments.

## Methods

### Data sources and study population

We conducted a retrospective, cross-sectional study utilizing the 2022 Centers for Medicare and Medicaid Services (CMS) Inpatient Provider Utilization and Payment Data. The analysis isolated hospital-level records utilizing the specific MS-DRG assigned to extracorporeal membrane oxygenation or tracheostomy with mechanical ventilation >96 hours (MS-DRG 003). To contextualize hospital reimbursement within local economic conditions, CMS records were linked to the 2023 Bureau of Economic Analysis (BEA) estimates for Metropolitan Statistical Area (MSA) Gross Domestic Product.

### Statistical analysis

For each hospital, the average Medicare payment per discharge, average submitted covered charge, average total payment amount, and average Medicare payment amount were extracted. This data was aggregated to the state level to facilitate broad geographic comparison. One-way analysis of variance (ANOVA) was utilized to assess the significance of geographic variation across states. Pearson correlation testing was used to evaluate the relationship between MSA GDP and hospital-level average payments per discharge. Additional Pearson correlation analyses evaluated the relationships between submitted covered charges and actualized Medicare and total payments. A *p*-value of <0.05 was considered statistically significant.

## Results

### Geographic variation in reimbursement

**Figure 1:**
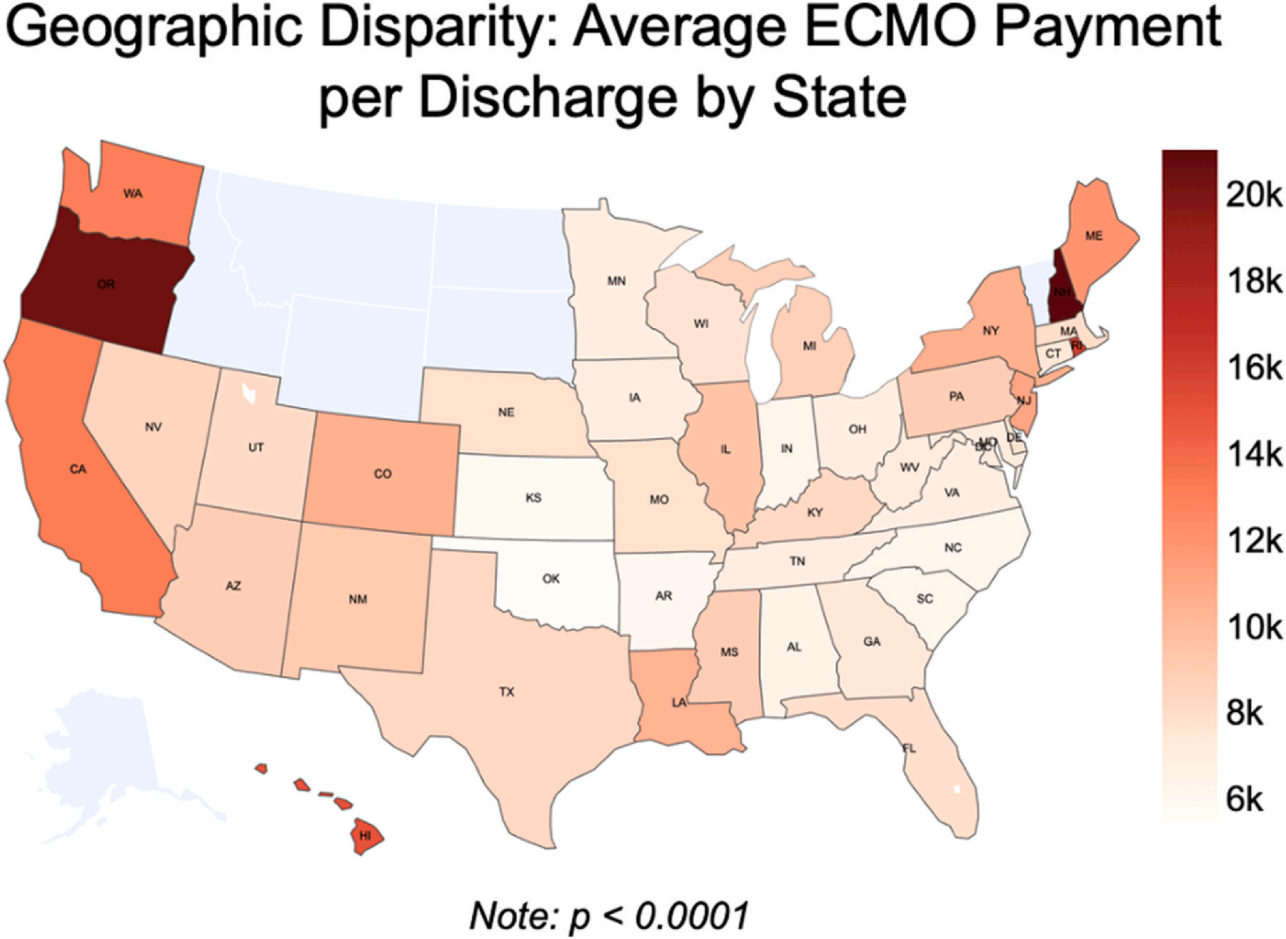
US map displaying geographic disparities in ECMO payments per discharge. ECMO, extracorporeal membrane oxygenation.

Analysis of the 2022 CMS inpatient cohort revealed significant geographic disparities in ECMO reimbursement. Substantial variation was observed across states in average payment per discharge (Figure 1), average submitted covered charge, average total payment amount, and average Medicare payment amount (all *p* < 0.0001).

**Figure 2:**
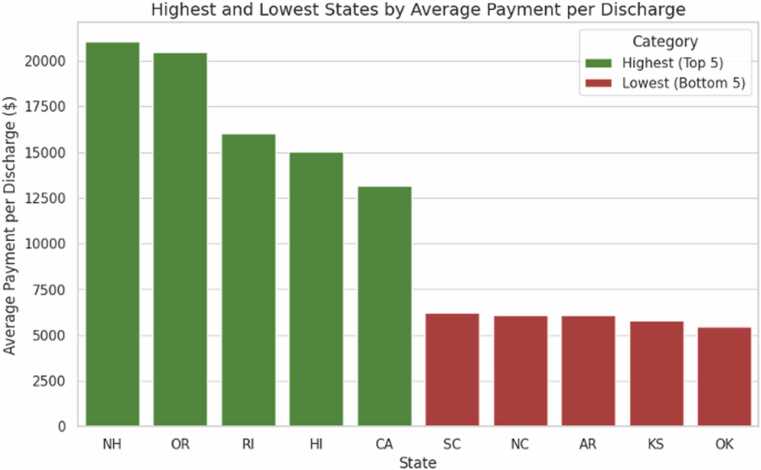
Mean medicare payment per discharge for ECMO in states with highest and lowest payments. ECMO, extracorporeal membrane oxygenation.

When evaluating the mean Medicare payment per discharge by state, a greater than threefold difference was identified between the highest- and lowest-reimbursed regions. The highest reimbursements were localized to New Hampshire ($21,045), Oregon ($20,460), and Rhode Island ($16,020). Conversely, the lowest average payments per discharge were in Oklahoma ($5,487), Kansas ($5,811), and Arkansas ($6,067) (Table 1, Figure 2).

**Table 1:** Mean Medicare Payment per Discharge for ECMO in States with Highest and Lowest Payments

**State**	**Total discharges (*n*)**	**Average submitted covered charges (USD)**	**Average total payment amount (USD)**	**Mean medicare payment per discharge (USD)**
*Highest Payments*				
New Hampshire	11	608,728	249,932	21,045
Oregon	28	821,062	299,405	20,460
Rhode Island	15	775,893	268,503	16,020
*Lowest Payments*				
Arkansas	67	483,526	129,860	6,067
Kansas	24	831,780	166,632	5,811
Oklahoma	139	824,168	151,970	5,487

ECMO, extracorporeal membrane oxygenation.

### Impact of regional economic performance

**Figure 3:**
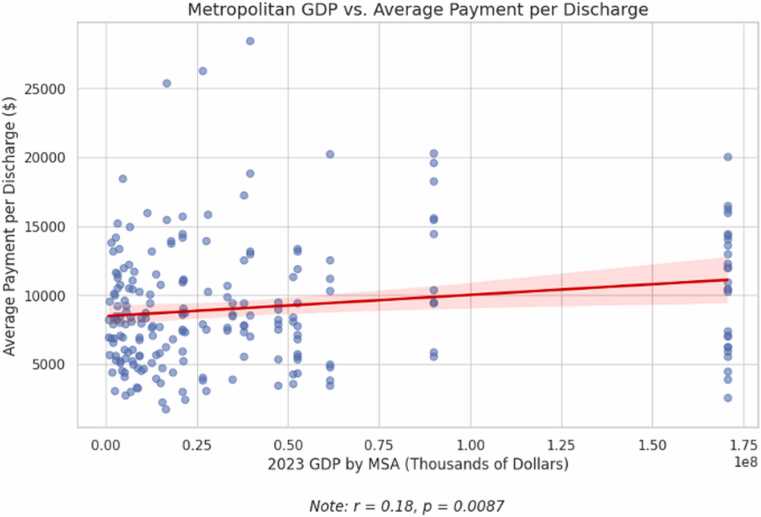
Relationship between metropolitan GDP and average payment per discharge for ECMO. Shaded area represents 95% confidence interval for line of best fit. ECMO, extracorporeal membrane oxygenation; GDP, gross domestic product.

Across the 213 hospital records successfully linked to MSA GDP data, regional economic performance demonstrated a weak but statistically significant positive correlation with average payment per discharge (*r* = 0.18, *R*² = 0.032, *p* = 0.0087) (Figure 3).

### Hospital billing practices vs realized payments

**Figure 4:**
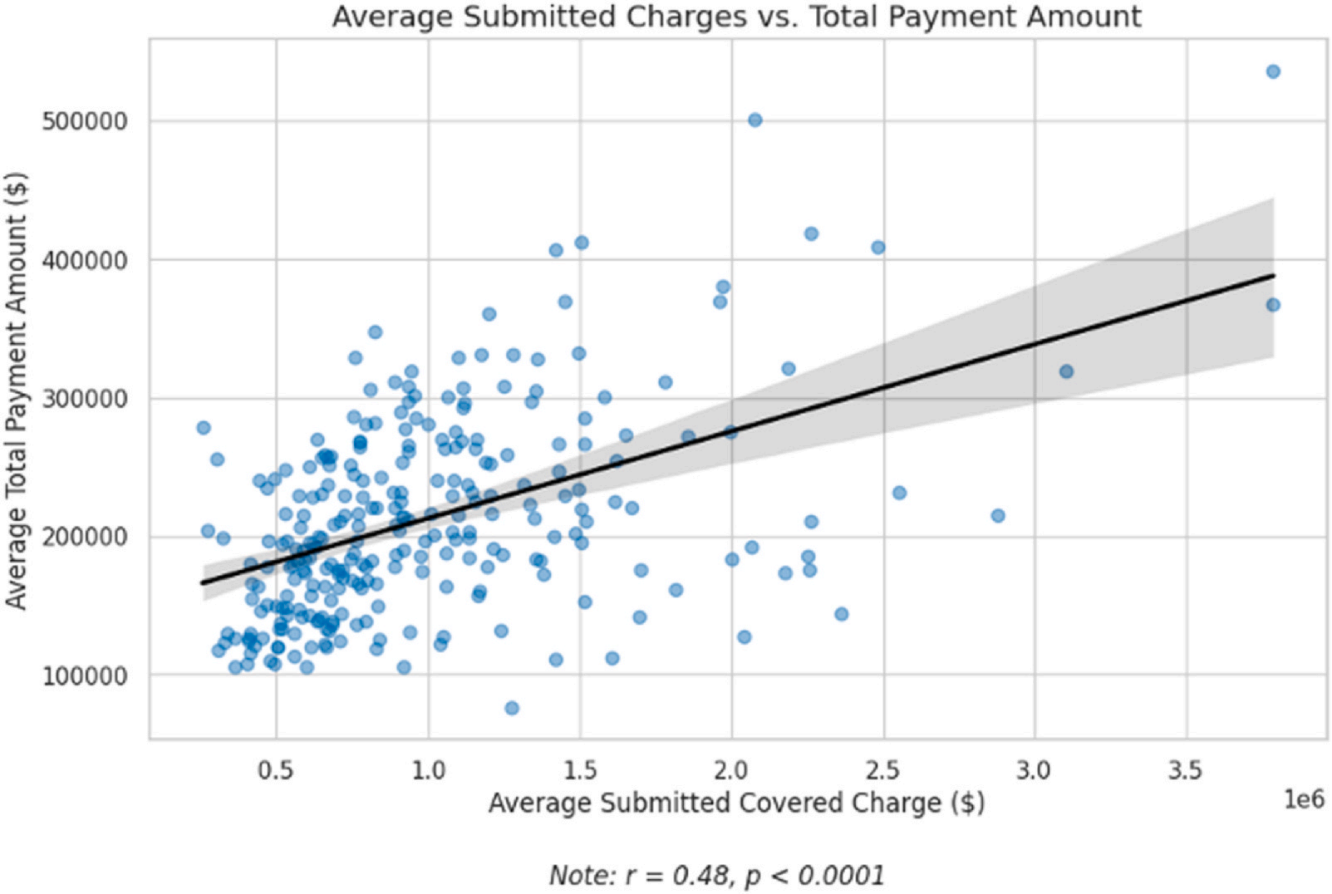
Relationship between average submitted charges and total payment amount for ECMO. Shaded area represents 95% confidence interval for line of best fit. ECMO, extracorporeal membrane oxygenation.

**Figure 5:**
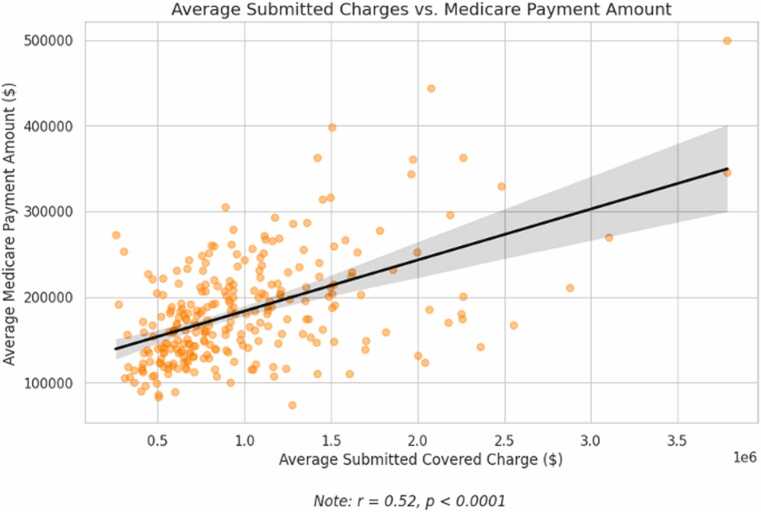
Relationship between average submitted charges and medicare payment amount for ECMO. Shaded area represents 95% confidence interval for line of best fit. ECMO, extracorporeal membrane oxygenation.

Hospital billing behaviors demonstrated a more robust relationship with reimbursement outcomes. Average submitted covered charges were moderately correlated with total payment amounts (*r* = 0.48, *p* < 0.0001) (Figure 4). Similarly, submitted charges exhibited a moderate correlation with realized Medicare payment amounts (*r* = 0.52, *p* < 0.0001) (Figure 5).

## Discussion

The financial viability of advanced cardiopulmonary support programs relies heavily on equitable and predictable reimbursement. The principal finding of this study is the persistence of extreme geographic variation in U.S. ECMO reimbursement, characterized by a more than 383% variance in Medicare payments per discharge between the highest- and lowest-paying states.

Importantly, our analysis demonstrates that regional economic health, measured by MSA GDP, explains only a small fraction of this variance (*r* = 0.18). While higher-GDP regions were loosely associated with slightly higher reimbursements, the low magnitude of the correlation indicates that local economic health is not the primary driver of the observed geographic disparities. Instead, structural factors, such as institutional billing practices, case mix, specialized coding utilization, and hospital-specific cost-to-charge ratios, likely contribute to the observed variation. The moderate correlation between submitted covered charges and realized Medicare payments (*r* = 0.52) suggests an association where institutions utilizing cost structures that generate higher submitted charges capture proportionally higher reimbursements. However, these descriptive findings should be distinguished from proven mechanistic hypotheses. GDP is an imperfect surrogate for geographic cost intensity, as it reflects economic output rather than healthcare-specific cost structures. Furthermore, standard Medicare reimbursement methodology involves critical adjustments not captured by GDP, including wage index modifiers, Indirect Medical Education (IME) payments, Disproportionate Share Hospital (DSH) distributions, and outlier payments, all of which substantially influence final reimbursement.

These findings echo concerns raised within the broader cardiothoracic surgery community regarding the endangered state of Medicare reimbursement frameworks.^4^ As the applications of ECMO broaden,^1^, ^2^ the current MS-DRG payment structure may insufficiently account for patient complexity, leading to disproportionately low financial support for hospitals in historically lower-billing states.^3^

## Limitations

This study is limited by its reliance on the CMS Medicare inpatient dataset, which may not accurately reflect private insurance reimbursement landscapes. Furthermore, MS-DRG data does not offer granular insight into important confounding variables that influence actual institutional costs and billing behaviors. These unmeasured variables include specific ECMO indication (veno-arterial vs veno-venous), duration of support, utilization as a bridge-to-transplant or bridge-to-left ventricular assist device, overall hospital ECMO volume, academic versus community hospital status, and individual patient complexity or comorbidity burdens. Additionally, aggregating our data at the state level may obscure important localized variation, as hospital density and discharge volumes fluctuate substantially within individual states.

## Conclusion

Substantial geographic variation in ECMO reimbursement persists across the United States. The modest correlation between metropolitan GDP and Medicare payments suggests that factors aside from local economic conditions contribute to these financial disparities. Further exploratory research into hospital-level factors and Medicare payment adjustments is necessary to fully understand these mechanisms. A reevaluation of reimbursement frameworks is vital to ensure sustainable access to high-level extracorporeal life support across all geographic regions.

## Declaration of Competing Interest

The authors declare that they have no known competing financial interests or personal relationships that could have appeared to influence the work reported in this paper.
